# A Rare Cause of Obstructive Sleep Apnea Syndrome: Retropharyngeal Lipoma

**DOI:** 10.1155/2017/2134362

**Published:** 2017-08-21

**Authors:** Okan Dilek, Omer Kaya, Cengiz Yilmaz, Gokhan Soker, Bozkurt Gulek, Mehmet Ali Akin

**Affiliations:** ^1^Mehmet Akif Inan Teaching and Research Hospital, Department of Radiology, University of Health Science, Sanliurfa, Turkey; ^2^Adana Numune Teaching and Research Hospital, Department of Radiology, University of Health Science, Adana, Turkey; ^3^Kozan State Hospital, Department of Radiology, Adana, Turkey

## Abstract

Lipoma is the most common benign mesenchymal neoplasm. About 16% of lipomas arise in the head and neck region, especially in the posterior neck. Large lipomas that originate from the retropharyngeal space may cause dyspnea, dysphagia, and snoring and occasionally may lead to obstructive sleep apnea syndrome (OSAS). Herein, we report a 45-year-old male patient with OSAS caused by a giant retropharyngeal lipoma with emphasis on CT findings.

## 1. Introduction

Lipoma is a common benign mesenchymal tumor. The head and neck region is a frequent site of involvement [1]. Large lipomas can cause dyspnea and obstructive sleep apnea syndrome [2]. In this report, we present a case with OSAS that was caused by a large retropharyngeal lipoma.

## 2. Case Presentation

**Figure 1:**
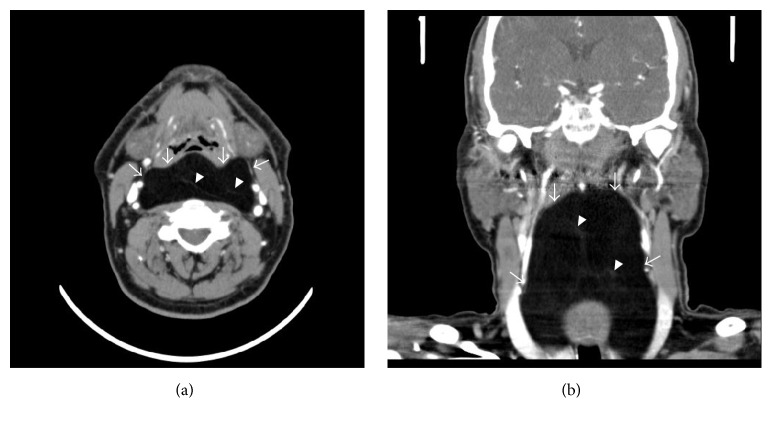
Axial (a) and coronal (b) CT images show a hypodense retropharyngeal mass measuring −80 HU, compatible with lipoma (arrows). Thin regular septations are also seen (arrowheads).

**Figure 2:**
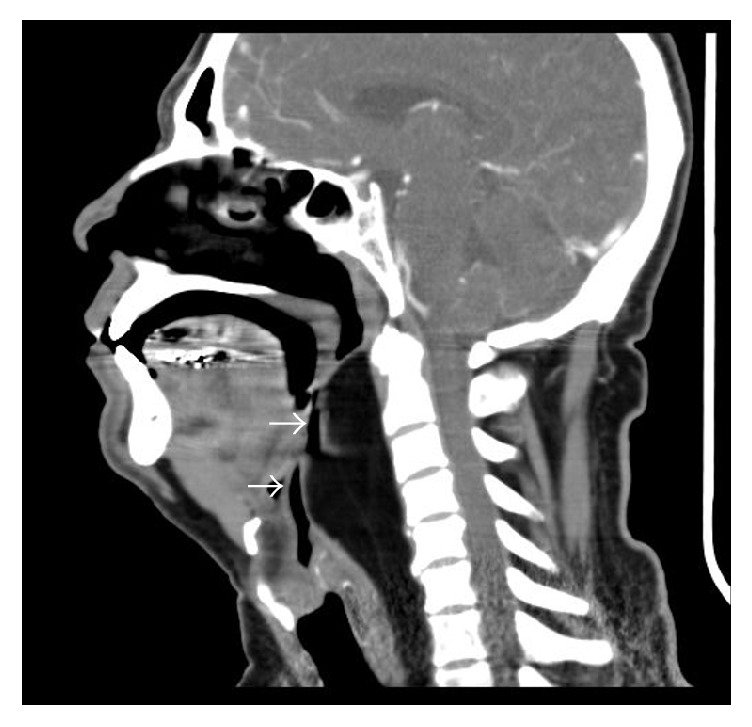
Sagittally reconstructed CT image shows narrowing of the airway (arrows) to a better extent.

A 45-year-old male patient presented to ear-nose-throat clinic with hoarseness, snoring, and drowning during sleep. Laryngoscopic examination revealed narrowing of the laryngeal column with an impression of external compression. Contrast-enhanced CT revealed a narrowed laryngopharyngeal airway and a giant retropharyngeal mass measuring approximately 4 × 8 × 12 cm. The mass was well defined with a few thin regular septae and did not enhance after injection of contrast material (Figures 1-2). The mass showed negative attenuation values (mean −80 HU) compatible with lipoma. The final diagnosis was OSAS due to giant retropharyngeal lipoma. Imaging follow-up was recommended for patient.

## 3. Discussion

Lipomas are the most common mesenchymal tumors and constitute about 16% of mesenchymal tumors. Approximately 25% of lipomas are located in the head and neck, especially in the posterior neck. Rarely, lipomas originate from the anterior neck and infratemporal space. Histologically, lipomas contain mature adipose tissue without cellular atypia and they have thin capsules [1].

Lipoma is seen in about 2% of people. Lesions are multiple in 5–15% of patients. They usually occur in the 5th and 6th decades. Lipomas are more frequently seen in obese patients and may increase in size with weight gain, although a decrease in size does not generally occur with weight loss [3]. Lipomas are slow-growing lesions and usually are not detectable until they grow to a large size. Large lipomas may compress neighbouring structures and may become symptomatic [4]. Patients with retropharyngeal lipomas often present with dyspnea or dysphagia. Occasionally, retropharyngeal lipomas may cause obstructive sleep apnea syndrome [2, 5].

At CT, lipomas appear as homogeneous hypodense masses with negative attenuation values between −50 and −150 HU. Lipomas do not enhance after injection of intravenous contrast material and may contain thin septae [6]. Hemorrhagic and fibrotic changes are rarely seen [4]. These findings are almost always diagnostic of benign lipomas and obviate biopsy to establish the diagnosis, as it was in our case.

Care should be taken not to mistake low grade liposarcomas for benign lipomas. At CT, low grade liposarcomas generally appear heterogeneous with thickened septae and mild enhancement [7, 8]. Lipoma variants are also described, such as fibrolipoma, osteolipoma, chondroid lipoma, intramuscular lipoma, pleomorphic lipoma, and lipoblastoma. They can be distinguished by clinical findings, radiological findings, and microscopic appearance from simple lipomas. Radiologic differences include thick septa, hemorrhagic, calcific, muscle, or fibrous tissue content, and contrast enhancement [1].

Surgical intervention is reserved for large symptomatic lipomas. Although lipomas are benign masses, they have the potential to grow over time. Thus, imaging follow-up is required for lesions that are close to vital structures such as the trachea or pharynx, as it was in our case [3].

In conclusion, our case report shows that retropharyngeal lipomas should be included in the differential diagnosis of patients presenting with obstructive sleep apnea syndrome, especially when no laryngeal or pharyngeal pathology is found at endoscopic examination.
